# Progress in research on site-specific nutrient management for smallholder farmers in sub-Saharan Africa

**DOI:** 10.1016/j.fcr.2022.108503

**Published:** 2022-05-15

**Authors:** P. Chivenge, S. Zingore, K.S. Ezui, S. Njoroge, M.A. Bunquin, A. Dobermann, K. Saito

**Affiliations:** a African Plant Nutrition Institute, UM6P Experimental Farm, Benguérir 41350, Morocco; b African Plant Nutrition Institute, ICIPE Campus, Duduville – Kasarani, Thika Road, Nairobi, Kenya; c Analytical Services Laboratory, Department of Soil Science, Agricultural Systems Institute, College of Agriculture and Food Sciences, University of the Philippines, College, Los Baños, Laguna 4031, Philippines; d International Fertilizer Association (IFA), 49, Avenue d′Iena, 75116 Paris, France; e Africa Rice Center (AfricaRice), 01 B.P. 2551, Bouaké 01, Côte d′Ivoire

**Keywords:** Digital decision support tool, Spatial variability, Nutrient use efficiency, Rice, Maize, Cassava, Site-specific nutrient management

## Abstract

Increasing fertilizer access and use is an essential component for improving crop production and food security in sub-Saharan Africa (SSA). However, given the heterogeneous nature of smallholder farms, fertilizer application needs to be tailored to specific farming conditions to increase yield, profitability, and nutrient use efficiency. The site-specific nutrient management (SSNM) approach initially developed in the 1990 s for generating field-specific fertilizer recommendations for rice in Asia, has also been introduced to rice, maize and cassava cropping systems in SSA. The SSNM approach has been shown to increase yield, profitability, and nutrient use efficiency. Yield gains of rice and maize with SSNM in SSA were on average 24% and 69% when compared to the farmer practice, respectively, or 11% and 4% when compared to local blanket fertilizer recommendations. However, there is need for more extensive field evaluation to quantify the broader benefits of the SSNM approach in diverse farming systems and environments. Especially for rice, the SSNM approach should be expanded to rainfed systems, which are dominant in SSA and further developed to take into account soil texture and soil water availability. Digital decision support tools such as RiceAdvice and Nutrient Expert can enable wider dissemination of locally relevant SSNM recommendations to reach large numbers of farmers at scale. One of the major limitations of the currently available SSNM decision support tools is the requirement of acquiring a significant amount of farm-specific information needed to formulate SSNM recommendations. The scaling potential of SSNM will be greatly enhanced by integration with other agronomic advisory platforms and seamless integration of digital soil, climate and crop information to improve predictions of SSNM recommendations with reduced need for on-farm data collection. Uncertainty should also be included in future solutions, primarily to also better account for varying prices and economic outcomes.

## Highlights

• The SSNM approach was adapted for rice, maize and cassava production systems in SSA.
• The SSNM approach increased yield, profitability, and nutrient use efficiency.
• SSNM had 24% and 69% higher yield than the farmer practice for rice and maize.
• Rice and maize yields were 11% and 4% higher in SSNM than local recommendations.
• Integration with other digital platforms and databases can improve SSNM tools.

## 1 Introduction

Sub-Saharan Africa (SSA) is the region most affected by food insecurity, with about 30% of the population experiencing recurrent food shortages due to persistently low yields (Van Ittersum et al., 2016). Crop yields in SSA have lagged behind other regions, mainly due to low soil fertility (Sanchez, 2002, Crawford and Jayne, 2010, Saito et al., 2019), as a result of low use of fertilizer and other soil amendments (Heffer et al., 2017, Vanlauwe and Dobermann, 2020). While yields of major cereal crops exceeding 6 Mg ha^−1^ have been attained on experimental stations, yields rarely exceed 3 Mg ha^−1^ on smallholder farmers’ fields in rainfed conditions (Bationo et al., 2007, Niang et al., 2017). There remains a large exploitable yield gap between attainable yield and farmers actual yields for the major cereal crops (Van Ittersum et al., 2016, Dossou-Yovo et al., 2020). While improved, high-yielding varieties of major cereals have been introduced in smallholder farming systems in SSA (Futakuchi et al., 2021), their yield potential has not been realized due to limited use of fertilizers, lack of irrigation, and other agronomic constraints (Cassman and Grassini, 2013). Although many smallholder farmers in SSA appreciate the role of fertilizers in crop production, fertilizer use has remained low due to a myriad of factors such as its high cost compared to other regions, lack of cash, poor market linkages, or variable returns to fertilizer use (Chianu and Mairura, 2012). Huge variation in fertilizer prices exists across the whole continent, and both fertilizer and grain prices constrain food production in SSA, often providing insufficient economic incentives to do better (Bonilla-Cedrez et al., 2021).

Increased fertilizer use, coupled with increased access to irrigation, mechanization, adoption of improved crop varieties and other inputs were the key drivers of crop production intensification in many world regions after World War II. However, the specific solutions and productivity gains were variable across regions. While the increased productivity spared the conversion of new land for agriculture in other regions, in SSA crop production increases have mostly been achieved through area expansion with small increases in productivity per unit area (Arouna et al., 2021a). Sub-Saharan Africa has experienced persistently low annual growth in agricultural total factor productivity (less then 1% for most of the past 60 years), making it food insecure and increasingly dependent on food imports (Keith, 2015).

Lessons from other global regions clearly show that increasing fertilizer use in SSA is imperative in order to intensify crop production and overcome the prevalent food and nutrition insecurity (Tsujimoto et al., 2019). In a bid to improve crop production through increased use of fertilizers and other modern inputs, the former UN Secretary General, Kofi Anan called for an ‘African Green Revolution’ in 2004. Despite some investments to increase the availability and use of fertilizers in SSA, mainly through input subsidies (Jayne et al., 2013), fertilizer use has remained around 16 kg nutrients ha^−1^, way below the 2006 Africa Fertilizer Summit target of 50 kg ha^−1^ by 2016 (Vanlauwe and Dobermann, 2020).

The yield benefits from increased fertilizer use in SSA have often been inconsistent and this has also brought into question the economic viability of subsidy programs (Jayne et al., 2013, Barrett and Bevis, 2015). Responses to fertilizer application may also be low due to generally poor agronomic management practices (Saito et al., 2019, Ravensbergen et al., 2021). In regions that achieved substantial increases in fertilizer use during the Green Revolution period, the early years were typically associated with a rapid increase in crop yields, followed by slower relative yield growth in subsequent decades. It also became known over time that the prevailing blanket fertilizer recommendations for fertilizer use failed to provide the correct nutrient balance, with excess N application in many cases leading to low nitrogen (N) use efficiency, whereas nutrients such as phosphorus (P) or potassium (K) were lacking. In the 1990 s, research done in lowland rice systems of Asia revealed a much higher degree of field-to-field variability in soil nutrient supply and response to fertilizer applications than previously assumed. Based on the lessons from other regions, it is evident that improving crop production in SSA will require increased fertilizer use, while ensuring that critical issues of tailoring fertilizer recommendations to physical and socio-economic environments are appropriately addressed.

Besides numerous other factors influencing farmers’ decision-making, fertilizer use across SSA is mostly still guided by general fertilizer recommendations for crops and regions. There is, however, often inadequate documentation of the scientific basis used to develop such (uniform) blanket fertilizer recommendations. Some reports suggest that blanket fertilizer recommendations were based on crop yield response to fertilizers for specific varieties at specific locations, which is then extrapolated to other locations and crops, with or without further field evaluation (Chapoto et al., 2016). In a more recent and better documented example, on-farm and on-station field trials were conducted, followed by fitting mathematical functions for grain yield response to applied nutrients in order to determine economical optimum rates of fertilizer application for a range of input and output prizes scenarios (Liben et al., 2020). An attempt was then also made to extrapolate such response functions to other regions through climate-based technology extrapolation domains (Liben et al., 2021).

The main drawback of blanket fertilizer recommendations is their failure to account for the high spatial soil fertility variability that is common in smallholder farming systems (Smaling et al., 1997, Njoroge et al., 2017, Niang et al., 2018, Ichami et al., 2020). Such variability has been linked to inherent soil fertility differences (Bationo et al., 2012, Niang et al., 2017), or differences induced by management practices (Wopereis et al., 1999, Zingore et al., 2007, Tittonell et al., 2008). Consequently, with blanket fertilizer recommendations, nutrients can be applied in excess or inadequately for different locations, reducing the efficient utilization of applied nutrients. Excess application of nutrients can lead to nutrient leakages to the environment resulting in adverse environmental impacts such as pollution of surface and groundwaters. Another problem is that some soils require that other constraints be addressed first, before any yield responses to nutrient additions are observed (Zingore et al., 2007, Vanlauwe et al., 2011, Tsujimoto et al., 2019). For example, increasing soil organic matter was observed to be prerequisite for achieving viable yield responses in degraded soils (Marenya and Barrett, 2009). Likewise, liming acidic soils is a pre-requisite for obtaining profitable response to fertilizer applications, but the requirements for that are also site-specific (Hijbeek et al., 2021). Alternative integrated soil fertility management practices are also needed to restore depleted soils, and ensure long-term environmental and crop production sustainability. In rainfed lowland rice farming systems, yield response to fertilizer application is usually poor in sandy soils (Niang et al., 2018, Asai et al., 2021) highlighting the need for customized fertilizer recommendations.

An opportunity exists to increase nutrient use efficiency through balanced and more precise plant nutrition to enhance food production and protect environmental resources. Optimizing crop productivity will depend on flexible fertilizer management practices tailored to spatial variation to ensure optimum nutrient use and avoid under- or over-supply of nutrients in crop fields. One of the solutions is the site-specific nutrient management (SSNM) approach developed in the 1990 s for smallholder rice production systems in Asia (Dobermann and White, 1998) and introduced to other crops and to SSA during the past 20 years.

Here we review the progress of this research in SSA and provide perspectives for future to sustainably increase crop productivity, profitability, and nutrient use efficiency through SSNM in SSA. The specific objectives of this review paper were to (i) provide a historical perspective on the development and application of SSNM for rice, and how this was extended to maize and cassava, (ii) review the status of field research, evaluation and dissemination of SSNM, and (iii) discuss needs for future work in SSA. Aligning with this special issue entitled by “Sustainable productivity enhancement of rice-based farming systems in Africa" in Field Crops Research, we give special attention to rice, which formed the basis of developing the SSNM approach in Asia and was also the first crop used to adapt the concept in SSA. It is also noted that while SSA contributes only about 4.5% of global rice production (GriSP, 2013), consumption demand is increasing faster than in any other world regions, outpacing productivity growth (Arouna et al., 2021a). Average rice yields in SSA are 2 Mg ha^−1^, which is less than half the global average, and the region relies on imports for more than half of its rice consumption. The low yield is mainly constrained by sub-optimum agronomic practices, including fertilizer management (Ibrahim et al., 2021, Saito et al., 2019, Saito et al., 2021). We also included SSNM for maize and cassava in this paper, as they are also widely produced in SSA, and often grown by the same rice farmers. Cross-learning across different crops allows to improve SSNM and its adoption in SSA.

## 2 Development of the SSNM approach

**Fig. 1:**
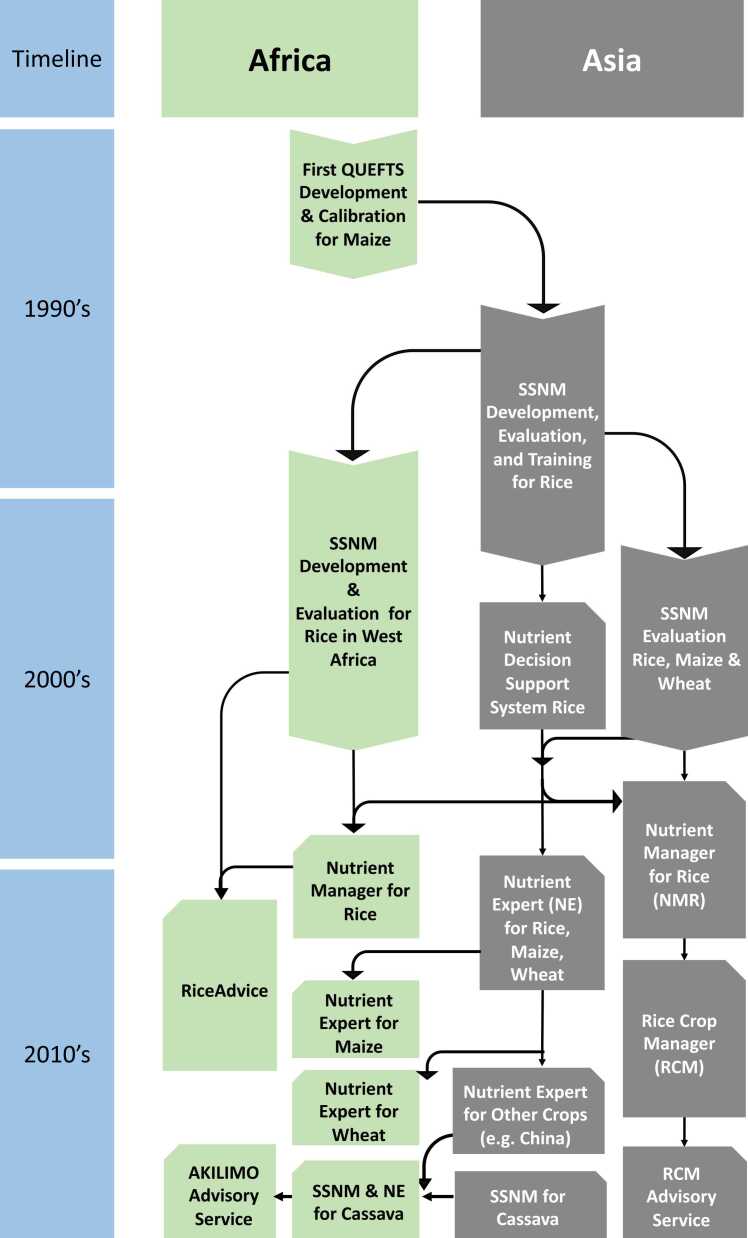
The evolution of site-specific nutrient management (SSNM), including the development of digital decision support tools in Africa and Asia.

The SSNM approach is a dynamic, plant-based, field- and season-specific nutrient management approach that aims to synchronize nutrient supply and demand according to differences in crop requirements, indigenous nutrient supply (INS), and nutrient recovery from fertilizer and other sources (Dobermann and White, 1998, Dobermann et al., 2002, Dobermann et al., 2004). It was developed by researchers of the International Rice Research Institute (IRRI) and their national partners in Asia in the 1990′s to address spatial variability in soil fertility and response to fertilizer application in smallholder rice farming systems (Fig. 1). It provided a new scientific basis for generating field-specific recommendations for fertilizer N, P and K (Dobermann et al., 2004), which could be tailored to different environments. It was conceptualized from the scientific principles of the Quantitative Evaluation of the Fertility of Tropical Soils (QUEFTS) model, which was initially developed using maize data from on-farm trials in Kenya (Janssen et al., 1990). With SSNM, the fertilizer nutrient requirements for a specific field are calculated from the difference between the total amount of nutrient required by the crop to achieve a given target yield and the INS (Witt and Dobermann, 2004), which reflects the amount of a particular nutrient (N, P or K) available from the soil, crop residues, irrigation water or biological N fixation during one crop cycle (Dobermann et al., 2003a). Generally, nutrients available in the soil are not in balanced proportions as required by different crops, making blanket recommendations inadequate. Moreover, particularly in irrigated rice, nutrient inputs from sources other than soil were found to be important, but were generally not included when making general fertilizer recommendations.

Soil testing is not widely available in smallholder farming systems and has general limitations in assessing the effective nutrient supplying capacity (Dobermann et al., 2003a, Schut and Giller, 2020). Consequently, in SSNM the INSs were estimated based on plant nutrient uptake or grain yield in nutrient omission trials, which involve growing a crop with an adequate supply of nutrients minus the one whose supplying capacity is being determined. For example, the indigenous supply of N can be measured as plant N accumulation in an N omission plot, which is sufficiently supplied with fertilizer P and K (and other nutrients if needed), but no fertilizer N (Dobermann et al., 2003a). Typical nutrient omission trials comprise of a set of five treatments that include (i) control (no nutrients added), (ii) PK (N omitted), (iii) NK (P omitted), (iv) NP (K omitted), and (v) NPK. Estimating INS in that manner is fundamentally different from soil testing approaches for deriving fertilizer needs, which are predominant in many other world regions, particularly those with established commercial farms and support services. It provides a direct, quantitative estimate of nutrient supply (e.g. kg ha^−1^) and thus allows the calculation of the specific amounts of additional nutrients needed to obtain a certain crop yield. With SSNM, fertilizer application is timed to match supply and peak demand periods for each nutrient for a particular crop, increasing nutrient uptake and nutrient use efficiency.

The key thrust of SSNM is to increase crop yields and profitability for the farmer by improving nutrient use efficiency. Field- and season-specific fertilizer N, P and K requirements for a particular crop are calculated before the start of the season. Fertilizer P and K requirements are determined to avoid yield loss due to deficiencies and avoid mining the soil nutrients while ensuring profitability (Buresh et al., 2010). For N fertilizer, SSNM calculates the optimum amount to be applied, and further determines the appropriate distribution through the growing season to match the peak demand for N by the crop, depending on the needs of specific crop types and varieties at key growth stages (Dobermann et al., 2002, Peng et al., 2010). Additional tools such as leaf color charts can be used for further in-season adjustment of the projected N applications, thus enabling further fine-tuning in response to the actual growth conditions (Fairhurst et al., 2007).

The SSNM approach originally developed for rice across Asia was refined through years of research (Dobermann et al., 2002, Dobermann et al., 2004), and has been applied to wheat (Khurana et al., 2008) and maize (Pasuquin et al., 2014) and other crops in Asia. In China, a digital platform, Nutrient Expert (http://www.nutrientexpert.cn/), provides SSNM solutions for 23 different crops, including grain crops, cash crops, vegetables, and fruits. The approach has also been introduced to SSA for rice (Haefele et al., 2003, Saito et al., 2015, Saito et al., 2019), maize (Rurinda et al., 2020) and cassava (Ezui et al., 2016).

## 3 SSNM R4D for rice in SSA

### 3.1 1990s and 2000s

Although the pioneering work on the development of the QUEFTS model was done for maize in East Africa (Janssen et al., 1990) (Fig. 1), research on SSNM in SSA, similar to Asia, was started in rice cropping systems. During the same time that IRRI developed the SSNM approach in the 1990s and early 2000s, scientists at the West Africa Rice Development Association (WARDA; now Africa Rice Center [AfricaRice]) learned about the SSNM approaches from colleagues at IRRI and developed variety-, site- and season-specific nutrient management recommendations for irrigated lowland rice in Burkina Faso, Mali, and Senegal (Haefele et al., 2003, Haefele and Wopereis, 2004, Segda et al., 2005). Although the nutrient management interventions for irrigated lowland rice in those countries were not termed SSNM, they in fact followed the same processes and principles as SSNM approaches for rice in Asia. This was motivated by the need to reduce the large rice yield gaps between actual and attainable yield that were largely attributed to suboptimal nutrient management (Wopereis et al., 1999). At the time, only blanket fertilizer recommendations were available to rice farmers in West Africa, despite the large spatial variability in soil fertility and weather conditions. The variety-, site- and season-specific nutrient management recommendations allowed rice farmers to manage nutrients tailored for individual fields.

Two simulation models, the dynamic ecophysiological ORYZA_S and RIDEV were used to determine potential yield levels as a function of sowing date, location, and variety choice (Dingkuhn, 1995, Dingkuhn and Sow, 1997, van Oort and Dingkuhn, 2021). For example, in Office du Niger in Mali, the ORYZA_S model was used to determine potential yield, based on weather conditions, variety and sowing date (Haefele et al., 2003). This potential yield was then used in the static FERRIZ model, which was based on the QUEFTS model, together with on-farm data on recovery efficiency of applied N, P and K, indigenous soil N, P and K supplies, and maximum N, P and K accumulation and dilution in rice dry matter. The resulting outputs were optimal fertilizer rates necessary to obtain specific target yields. Integrating prevailing fertilizer and paddy prices into the analyses allowed coupling agronomic evaluation with an economic analysis module to derive economically optimal fertilizer management options. In the last step, the dynamic decision tool RIDEV was used to simulate optimal timing of different agronomic management actions, such as fertilizer application, weeding and harvest. This approach showed that (i) prevailing uniform recommendations for the wet season performed well except on low-K soils where the application of K was profitable; and (ii) lowering the fertilizer doses to the lower potential yield in the dry season reduced costs and risk without reducing profitability. Based on this analysis, when only focusing on the existing recommendations, there was scope to adjust nutrient application for the wet and dry seasons, keeping fertilizer costs and risk low, and maintaining net benefits close to optimal. This approach is particularly useful in SSA where the use of fertilizers is variable but has remained very low on average (Heffer et al., 2017, Vanlauwe and Dobermann, 2020).

In a separate study in Bagré irrigation scheme in Burkina Faso, Segda et al. (2005) used a combination of RIDEV and FERRIZ simulation models together with data obtained in field trials to develop alternative fertilizer recommendations. Existing fertilizer recommendations in Bagré were 82 or 105 kg N ha^−1^ for the wet and dry season, respectively, plus 31 kg P ha^−1^ and 30 kg K ha^−1^. RIDEV was used to optimize timing of sowing to avoid cold-induced sterility and timing of N fertilizer applications. FERRIZ was used to determine alternative fertilizer recommendations, based on estimations of indigenous N, P, and K supplies, potential yield, internal N, P and K efficiency of rice, fertilizer N, P and K recovery fractions, and fertilizer and rice prices. Simulations suggested the need to decrease P and K doses to 21 and 20 kg ha^−1^, respectively, but increasing the N dose to 116 and 139 kg ha^−1^ for a target yield of 8 and 9 Mg ha^−1^ for the wet and dry season, respectively. The alternative fertilizer recommendation aimed to keep a neutral P balance, but a negative K balance was tolerated based on the high indigenous soil K supply. Compared to existing recommendations, yield gains of up to 0.5 Mg ha^−1^ were simulated at equal fertilizer costs. These yield gains were exceeded in farmers’ fields during four consecutive growing seasons. Alternative fertilizer recommendations increased gross returns above fertilizer costs by an average of about USD 160 per season compared to both farmers’ practice and existing recommendations. Although farmers did not follow the alternative recommendations precisely, they adopted the guiding principle from the study – to apply more urea and less NPK compound fertilizer (Segda et al., 2010).

Based on the approach used in Burkina Faso and other studies, recommended nutrient management practices for irrigated lowland rice in Senegal River Valley were modified and recommended N application rate ranged from 133 to 179 kg ha^−1^, applied as di-ammonium phosphate (DAP) at around sowing or transplanting, and three splits of urea at early tillering, panicle initiation, and booting (Saito et al., 2015). Timing was guided by RIDEV depending on weather, variety and sowing time. Differences in application rates depended on season and agro-ecological zone. The recommended rate was higher in the dry than wet season, and higher in the Senegal River delta than in the middle valley, where extreme temperatures tend to occur and affect potential yield (Haefele and Wopereis, 2004). Recommended P and K rates were 20 and 0 kg ha^−1^, respectively. Potassium fertilizer application has not been recommended because K inputs from irrigation water and from dust depositions are considered sufficient to meet crop demand, except when the target yield was more than 8 Mg ha^−1^ and farmers practiced double cropping.

### 3.2 Progress in the 2010s

The SSNM recommendations described above were effective in increasing yields and profit for farmers despite not taking into account previous management such as preceding crop and its management, or the financial limitations of farmers (Saito et al., 2015). Nonetheless, there remained challenges for dissemination to individual smallholder farmers. The calculations of nutrient requirements using SSNM principles are knowledge intensive such that most of local public extension workers had limited capacity to generate field-specific recommendations in the early 2000 s, even in Asia. Advances in information technology and mobile communications have now made it possible to develop tools that enable extension workers and farmers to generate and disseminate SSNM recommendations using a smartphone or tablet. A first step in that direction had already been made in 2005, when a Nutrient Decision Support System (NuDSS) software was released for irrigated rice in Asia (Witt et al., 2005). Besides the standard SSNM calculations, it included a simple model for estimating rice yield potential as well as modules for optimizing split N applications, fertilizer choices and profit. Its main drawback was that it was too complex for practical use by extension workers or farmers. Therefore, around 2008, IRRI began to develop a simplified decision-support tool based on SSNM principles, the Nutrient Manager for Rice (NMR). It first included web versions and a mobile phone-based service (interactive voice response through a toll-free number to dial), and evolved further towards a family of smartphone apps branded as Rice Crop Manager (RCM; http://cropmanager.irri.org) for different regions in Asia (Buresh et al., 2019). The key feature of this process was the systematic incorporation of other relevant local information, including advice on few other agronomic measures that are critical for nutrient response.

With successful testing and scaling of NMR in Asia, scientists from IRRI and AfricaRice jointly developed a NMR version for the Senegal River valley. NMR was accessed through the internet using a personal computer, smartphone, or tablet by agricultural extension officers or lead farmers. The recommendations were calculated based on farmers’ responses to questions about the agro-ecological or administrative zone of their field, rice variety, availability of irrigation water, previous crop and residue management, previous rice yield levels, and fertilizer use. The recommendations provided by NMR in 102 farmers’ fields in the Senegal River valley increased rice yields by 1.1–2.3 Mg ha^−1^ (equivalent to 19–36%) and profit by USD 586–1309 ha^−1^ (corresponding to 19–39%) compared with farmers’ usual fertilizer management practice (Saito et al., 2015).

Based on the successful testing of NMR, AfricaRice conducted nutrient omission trials on 30 sites in 17 countries in SSA (Saito et al., 2019), and developed a digital decision-support tool, RiceAdvice (https://www.riceadvice.info/en/). RiceAdvice is similar to NRM, but unlike NMR, it does not require internet access to generate nutrient management recommendations. RiceAdvice provides farmers with customized fertilizer recommendations based on their financial resources and/or target yield, which is based on the yield from the previous season (Saito and Sharma, 2018). By the end of 2020, some 100,000 recommendations from RiceAdvice had been generated for use by farmers in Burkina Faso, Mali, Nigeria, and Senegal. In assessing the impact of RiceAdvice in Kano State, Nigeria, Arouna et al. (2021b) showed that farmers who received only RiceAdvice recommendations increased their yields by 7% (250 kg ha^−1^) over the control, which translated into a 10% increase in profit (USD 120 ha^−1^). In contrast, farmers who received the RiceAdvice recommendations and were given the recommended fertilizers increased their yields by 20% (730 kg ha^−1^) and their profit by 23% (USD 275 ha^−1^). Thus, although RiceAdvice recommendations alone have a positive impact, improved access to inputs is required for precise adoption of recommended fertilizers and maximization of expected benefits, because smallholder farmers are generally resource constrained. Zossou et al. (2021) assessed farmers’ perceptions of RiceAdvice and its associated benefits in the same state and observed that by using RiceAdvice, more than 90% of farmers noted that the amount of nutrients applied can be reduced by more than 25% and about 84% of the farmers reported increased yield and income by over 25%. Given that generating RiceAdvice recommendations requires an Android-based phone or tablet, which are not available to all farmers, using RiceAdvice at scale requires strengthening public extension systems, developing appropriate business models that include private service providers, and including female service providers to reach female farmers (Zossou et al., 2021).

RiceAdvice has been evaluated and disseminated by AfricaRice with its partners, mostly in irrigated and favourable rainfed lowland rice systems. A recent field study (Niang et al., 2018) and a review paper in this special issue (Asai et al., 2021) clearly show the need to develop field-specific recommendations that take into account other biophysical factors, particularly in drought-prone rainfed systems. Niang et al. (2018) showed that year-to-year variation in rainfall and spatial variation in field water status strongly drive seasonal fluctuations in rice yield and yield response to N fertilizer application in rainfed systems in central Benin. Yield response to applied N tends to be less when water deficits are severe, while spatial variations in field water status are related to the sand content of soils. Thus, further studies to examine the linkage between farmers’ knowledge (of water status and soil texture), and field water status assessment, laboratory analysis (including soil sand content), and rice productivity, could help in developing a field-specific decision-support system (Saito et al., 2006). Furthermore, as water status in each field is affected by seasonal rainfall pattern and amount, reliable forecasting is needed to help farmers decide whether to apply fertilizer or not. In addition to bunding, used in some farmers’ fields in the area, other water conservation measures, such as mulching, land-leveling, or no tillage, should be evaluated. However, as heavy clay soils tend to have cracks when dry, bunding and no-till systems might not work in these conditions. In this special issue, Asai et al. (2021) conducted a meta-analysis of yield responses to mineral fertilizers in relation to biophysical factors for rainfed upland rice in SSA using Bayesian models. They found that N fertilizer effects on yield response were dependent on soil type, with poor or negative responses in low clay soil, especially under low precipitation conditions. These findings clearly indicate the need for considering soil type and expected precipitation in the development of SSNM approaches adapted to drought-prone environments in SSA.

### 3.3 SSNM research for maize in SSA

SSNM recommendations for maize were introduced to SSA together with a digital decision support tool, Nutrient Expert (NE; Fig. 1), initially developed in Asia (Pampolino et al., 2012). NE is based on the same principles and main algorithms that were used in the NuDSS and NMR software that had been developed earlier for rice, including key elements of QUEFTS. It was designed as a digital fertilizer advisory tool that can be accessed using a computer or smartphone. In NE, the fertilizer requirements for a field or location are estimated by using the same concept as SSNM for rice presented in Section 2.

As a first step, nutrient omission trials were conducted in hundreds of farmers’ fields, over two cropping seasons (2015 and 2016) in Nigeria (n = 423) and Ethiopia (n = 148), and one cropping season in Tanzania (2016–2017; n = 300), to calibrate NE for these environments (Rurinda et al., 2020). Following collection of relevant data from the nutrient omission trials, N, P and K yield response functions in NE were calibrated using country specific datasets and separate versions of NE calibrated for Nigeria, Ethiopia, and Tanzania, and used to generate SSNM recommendations for a broad range of yield response domains based on yield response data from the nutrient omission trials (Njoroge et al., 2017; Shehu et al., 2018). Additional trials to calibrate and validate NE have also been conducted in Zimbabwe, Kenya and Senegal.

Evaluation of NE performance against soil-test based and blanket recommendations in 58, 108, and 202 farmers’ fields in Tanzania, Ethiopia and Nigeria, respectively revealed positive agronomic and economic benefits of applying NE based recommendations (Rurinda et al., 2020). NE recommended lower fertilizer P and K application rates relative to soil-test based and blanket recommendations in Nigeria, and lower fertilizer P application rates relative to the blanket and soil-test based recommendations in Ethiopia and Tanzania, respectively. Despite these variations in nutrient recommendations, maize yields in Nigeria and Ethiopia were not significantly different among the three recommendation methods. Similar yields at lower P and K application rates with NE resulted in improvements in agronomic efficiencies of fertilizer P and K over the blanket recommendations in Nigeria, with an associated increase in net economic benefits from fertilizer use.

As part of SSNM dissemination within SSA through the Taking Maize Agronomy to Scale in Africa (TAMASA) project, a program to support large-scale dissemination of NE recommendations was implemented in Nigeria in a partnership between research, development, government extension services and the fertilizer industry. The program delivered recommendations to 20,000 farmers and results from an impact assessment study showed that NE recommendations led to yield improvement by 65% and income by 40% (Peter Craufurd, International Maize and Wheat Improvement Center, personal communication). It was also observed that 90% of the farmers covered by the program had no alternative sources of extension information, indicating a major role for tools such as NE in reducing the critical knowledge dissemination gap. Results from an impact assessment study that used a randomized control treatment approach indicate that smallholders’ access to SSNM advice increased maize yields by 0.2–0.4 Mg ha^−1^ ( 9–19%) over one to two years, resulting in increased profitability by USD 69 ha^−1^ (14%) (Oyinbo et al., 2021). Furthermore, the study shows that reducing farmers’ uncertainty by providing additional information on the variability in expected returns induced by price uncertainty resulted in gradual investments and expansion of fertilizer use compared to farmers who only received SSNM recommendations. Providing SSNM recommendations with additional information on variability on expected returns renders agricultural extension more effective.

### 3.4 SSNM research for cassava in SSA

The application of SSNM in cassava in SSA has been limited for a long time due to a general perception that cassava is mostly grown without or with low nutrient inputs (van Fermont, 2009). Moreover, cassava is known for its ability to grow and produce storage roots even under harsh growing conditions like drought, low soil fertility and acid soils, where other major food crops often fail (Hillocks, 2002). Hence, farmers often grow cassava with minimal or no nutrient applications, or apply fertilizers only to associated intercrops, such as maize. However, response of cassava to fertilizer is commonly observed (van Fermont, 2009). Compared with average yields under farmers practices of about 7.5 Mg ha^−1^ of fresh storage roots, yields of 90 Mg ha^−1^ (Adiele et al., 2020) can be achieved under favorable growing conditions with good agronomic practices and improved fertilizer management practices. However, blanket recommendations were inadequate given the diversity of soils and growing conditions within and between farms.

Recent progress in cassava growth modelling has led to the development of SSNM recommendations for cassava production. Mechanistic crop models such as LINTUL-CASSAVA (Light Interception and Utilization) (Ezui et al., 2018, Adiele et al., 2021a) and MANIHOT (Moreno-Cadena et al., 2021) were developed to simulate location-specific attainable and potential yields of cassava, and to define the best periods for planting and harvest of a given cultivar using historical weather data. Byju et al. (2016) and Ezui et al. (2016) adapted the QUEFTS model for Indian and African conditions, respectively, to estimate optimal requirements of N, P and K to achieve a certain target yield in a given location. In Ghana and Togo, using a balanced nutrition approach based on the QUEFTS model, higher nutrient use efficiency and cost-benefit ratios were achieved compared to blanket rates recommendations (Ezui et al., 2016). The African Cassava Agronomy Initiative Project led by the International Institute of Tropical Agriculture (IITA) in collaboration with the African Plant Nutrition Institute (APNI) followed the modelling framework to develop AKILIMO (https://www.akilimo.org/), a digital decision platform to aid dissemination of tailored agronomy advice in Nigeria and Tanzania.

### 3.5 An overview of benefits of SSNM in field experiments in SSA

Table 1 summarizes data from eight studies that evaluated SSNM in maize and rice cropping systems in SSA. It is important to note that the SSNM approach in rice systems was applied in irrigated lowland systems, which are characterized by better soils and relatively high fertilizer rates. SSNM studies for maize were only carried out in rainfed systems, which tend to have poorer soils and historically low nutrient input levels. On average, N fertilizer rates were 60 and 124 kg N ha^−1^ for the farmers’ practice in maize and rice systems, respectively (Table 1). These rates are relatively high and suggest that the sites chosen are not necessarily fully representative for the whole continent, considering that average fertilizer application rates are much lower than that (Vanlauwe and Dobermann, 2020). Overall, greater yield, N use efficiency and gross return above fertilizer cost benefits were observed when SSNM was compared with the farmer practice, but also when SSNM was compared with local blanket recommendations. Averaged across rice and maize, grain yield gains through SSNM were 1.3 and 0.4 Mg ha^−1^ when compared to farmer practice and local blanket fertilizer recommendations, respectively. This translated to 29% higher grain with SSNM than farmer practice and 8% than local blanket recommendation. It should be noted that studies comparing SSNM to the no input control were excluded, as it is expected that yield with fertilizer addition would be greater than the no input control. N use efficiency with SSNM was 28% higher than the farmer practice while it was 19% higher than with blanket fertilizer recommendations. Similalry, yield, nutrient use efficiency and profitability improvements were also observed for cassava in Ghana and Togo when SSNM recommendations were compared with the local blanket recommendations (Ezui et al., 2016). Recent work by Adiele et al. (2020), also revealed increased nutrient use efficiency and yield from improved management practices for cassava production in Nigeria. Unfortunately, apart from Ezui et al. (2016), there is no publication which assessed the performance of SSNM for cassava in SSA as yet.

**Table 1:** Summary of comparison of site-specific nutrient management (SSNM) to farmer practice (FFP) or state recommendation (state rec.).

Source/s	Country (# of sites)	Production system^β^	Season^α^	Decision tool^Ψ^	N rate (kg N ha^−1^)			P rate (kg P ha^−1^)			K rate (kg K ha^−1^)			Grain yield (Mg ha^−1^)			PFPN^γ^ (kg grain kg^−1^ N)			GRF^ℜ^ (USD ha^−1^)		
					SSNM	FFP	State rec.	SSNM	FFP	State rec.	SSNM	FFP	State rec.	SSNM	FFP	State rec.	SSNM	FFP	State rec.	SSNM	FFP	State rec.
**Rice**																						
Haefele et al. (2003)	Mali (3)	Irrigated	DS	FERRIZ + RIDEV	121		156	16		20	17		0	6.0		6.1	51		39	3223		3223
			WS	FERRIZ + RIDEV	172		128	33		20	141		0	7.9		7.0	47		55	4135		3757
Segda et al. (2005)	Burkina Faso (1)	Irrigated	DS	FERRIZ + RIDEV	139	81	105	21	15	31	20	15	30	6.6	5.2	5.9	57	63	56	3544	2779	3122
			WS	FERRIZ + RIDEV	116	76	82	21	17	31	20	16	30	6.3	5.2	5.5	54	68	67	3339	2804	2899
Saito et al. (2015)	Senegal (1)	Irrigated	DS	NE	141	150		18	14		34	0		6.9	5.8		49	38		3647	3061	
			WS	NE	118	161		14	24		10	0		8.7	6.4		74	40		4706	3397	
Africa Rice	Ghana (1)	Irrigated	DS & WS	RA	126	151	115	19	19	26	37	36	50	4.9	4.3	4.1	39	28	36	2575	2229	2121
**Maize**																						
Masego (2013)	South Africa (3)		WS	SPAD (timing)	26		58	42		42				4.0		3.8	171			1016		931
Oyinbo (2019)	Nigeria (17)			NE	133	60		25	8		48	15		5.3	2.1		40	34		1316	507	
Balemi and Rurinda (2020)	Ethiopia (7)	Rainfed	WS	NE	128		101	54		69	37		0	7.0		6.8	55		68	1737		1696
	Ethiopia (2)	Rainfed	WS	NE										6.8	5.1							
Rurinda et al. (2020)	Nigeria (14)	Rainfed	WS	NE	110		120	15		26	12		50	3.9		3.9	35		33	978		931
	Ethiopia (5)	Rainfed	WS	NE	120		111	22		30	26		0	6.8		6.8	57		61	1755		1765
	Tanzania (22)	Rainfed	WS	NE	100		100	12		9	12		0	2.7		2.7	27		27	657		669
**Treatment Comparisons**^**δ**^																						
SSNM vs. State for rice														6.3		5.7	50		50	3363		3024
SSNM vs. FFP for rice														5.6	4.6		48	41		3068	2447	
SSNM vs. State for maize														4.8		4.6	68		43	1554		1464
SSNM vs. FFP for maize														6.1	3.6		40	34		1316	507	
Overall														5.7	4.4	5.2	55	40	49	2373	2170	2111

Production system^β^: is based on soil-water conditions as influenced by the climate in the area where the crops were grown

Season^α^: is the period of the year during which the particular crop is cultivated

Decision tool^Ψ^: is the SSNM-based tool which provides fertilizer recommendation; RIDEV is used to simulate optimal timing of agronomic management actions; FERRIZ is based on QUEFTS model together with on-farm data; SPAD is SPAD chlorophyll meter; NE is Nutrient Expert; RA is RiceAdvise

PFPN^γ^: is partial factor of productivity of N; a measure of N use efficiency

GRF ^ℜ^: is gross return above fertilizer cost, which is calculated as GRF = Gross return - Total fertilizer cost

Treatment Comparisons^δ^: Means for comparisons between FFP or State rec. were not made across all the studies because some studies only compared SSNM to either FFP or State rec.

For rice systems, N fertilizer rates for SSNM in Saito et al. (2015) and AfricaRice (2016) were comparable to the local blanket recommendations but lower than the farmer practice. In some cases (Saito et al., 2015, Rurinda et al., 2020), K rates were higher with SSNM than farmer practice and the local recommendations for both rice and maize (Table 1). This is comparable to studies conducted with rice in Asia, that generally observed lower N but higher K rates were recommended with SSNM compared to the farmer practice, while average P rates were often similar (Dobermann et al., 2002, Xu et al., 2017, Chivenge et al., 2021a). In contrast N fertilizer rates with SSNM for maize were higher than both farmer practice and the local recommendations. Profitability of SSNM for maize was small compared to rice, particularly when SSNM was compared to blanket recommendations. The reasons for this could be related to difference in production systems between two crops, and the fact that yield gain with SSNM was higher in rice than maize. Rice was grown in irrigated lowlands, whereas maize was grown in rainfed systems. The difference in gross return above fertilizer cost between SSNM and blanket recommendations were marginal (6%; Table 1). However, data from the eight studies reviewed here suggest that grain yield, N use efficiency and profit gains can be realized when SSNM recommendations are followed, despite N rates with SSNM being higher or lower than the farmer practice or the blanket recommendations. This was probably because of an adjustment in aligning timing of nutrient application to coincide with periods of high nutrient demand.

The overview of the benefits of SSNM-based recommendations presented was limited to mean values for specific studies and regions, as we aimed to provide a high-level review of the evolution of the SSNM concept. The rigorous process of calibration and validation SSMN decision support tool is a crucial basis for a robust and effective system for generating nutrient management recommendations that consistently increase yields and economic benefits over farmer practice at the aggregate level. However, the performance of the SSNM recommendations may vary from field to field due to complex differences in growing conditions and management intensity at the farm scale. Several uncontrolled factors that can limit the effectiveness of SSMN recommendations within specific sites include (i) recommendations for the application of only N, P, K in sites where other nutrients are severely deficient; (ii) major changes in seasonal rainfall amount and distribution; (iii) reduced yields due to sub-optimal agronomic management in farmer-managed sites, e.g. planting time, crop establishment method, weed, pest and disease control,; and (iv) sites with severe non-nutrient limitations, e.g. soil acidity, that severely constraints crop yield response to nutrient application.

## 4 Discussion

Blanket fertilizer recommendations do not properly address the specific local biophysical conditions in agricultural production systems in SSA, often making them unprofitable or even a disincentive for smallholder farmers to use fertilizers (Kihara et al., 2016a). Previous on-farm studies have demonstrated the need to account for spatial heterogeneity in developing fertilizer recommendations (Vanlauwe et al., 2007, Zingore et al., 2007, Tittonell et al., 2008, Kihara et al., 2016b). The development of the QUEFTS model in SSA (Janssen et al., 1990) paved the way for the evolution of SSNM (Fig. 1) (Dobermann and White, 1998, Dobermann et al., 2002), which enables farmers to manage fertilizer application in accordance to spatial heterogeneity. The SSNM approach has evolved over 30 years of research and was refined across Asia and SSA (Fig. 1). This review highlights the potential contribution of SSNM in improving crop production in smallholder farming systems in SSA (Table 1).

Our findings complement a broader meta-analysis conducted for maize, rice and wheat across Asia and Africa, which showed greater yield benefits with SSNM in Africa and South Asia where farmer yields were low compared to South East and East Asia (Chivenge et al., 2021b). In East and South East Asia, where yields and fertilizer use are high, SSNM benefits compared to farmer practice were mostly observed through improved N use efficiency and more balanced crop nutrition. In the current study, we included comparisons to state or local recommendations, which were not included in the published study. Also, we included recent efforts made for development of SSNM for cassava. However, the studies evaluated here represent a small sample size, although SSNM has been researched and evaluated in the region for more about 20 years. This highlights the need to conduct more research and on-farm evaluation of SSNM to enable fine-tuning and refinement of SSNM under varying conditions and for different crops in the region. This will require using robust frameworks for geospatial characterization of yield gaps and diagnosis of yield limiting factors. Excellent methodologies have been developed for that in recent years (e.g. Rattalino et al., 2021) and it is now necessary to apply them more widely across SSA. This must also guide the representative selection of field sites used for collecting critical data and evaluating specific SSNM solutions (Ten Berge et al., 2019).

Field validation and dissemination of SSNM in SSA are still limited to few geographical locations and a few crops, and have generally lagged behind work done in Asia where SSNM was developed and has been refined across many countries and crops. For example, RiceAdvice has been developed, evaluated and disseminated for rice farming systems in West Africa only (Saito et al., 2015, Saito and Sharma, 2018). By the end of 2020, only 100,000 recommendations had been generated in four countries over a span of seven years, reaching only a small fraction of the rice farmers. For the similar period, 2.26 million (2013 to early 2020) and 230,000 (2017 to early 2020) recommendations had been generated in the Philippines and India respectively, where stronger public extension systems exist for scaling of digital advisory tools. NE for maize has only been calibrated for about six countries (Rurinda et al., 2020), despite maize being the main staple cereal crop in SSA. Dissemination of NE has been very limited to even fewer geographies. The impact assessment of the large scale dissemination of NE in Nigeria showed that most farmers do not have access to any form of extension services (Peter Craufurd, International Maize and Wheat Improvement Center, personal communication).

It is clear that SSNM research and dissemination efforts in the public sector need to be strengthened in SSA. Furthermore, business models that enhance the dissemination of SSNM and the associated digital tools together with private sector engagement are needed to promote farmer adoption. Strengthening the financial services to smallholder farmers and input supply systems, and reducing farmers’ uncertainty about variability of market prices could help scaling of SSNM. Arouna et al. (2021b) and Zossou et al. (2021) clearly highlighted greater benefits with SSNM when input availability was improved for resource-constrained smallholder farmers. However, most of the SSNM studies have largely focused on primary macronutrients; N, P and K, with limited inclusion of secondary macronutrients and micronutrients, which has caused mining of the omitted nutrients. Recent studies in SSA show increasing evidence that secondary macro- and micro-nutrients limit crop growth (Awio et al., 2021, Senthilkumar et al., 2021). Consequently, micronutrients need more attention as part of SSNM solutions and decision tools. This will also contribute to improving human nutrition.

The relatively high occurrence soils with peculiar constraints such as soil acidity, Mn and Al toxicity, soil erosion, soil crusts that affect germination and emergence, is a major challenge for developing SSNM based digital decision tools in SSA. The effect of such soil related constraints on crop yields is yet to be well captured in most available digital decision support tools (MacCarthy et al., 2015), which limits the applicability of such tools. Non-responsiveness of maize and soybean to fertilizer applications in African smallholder farms has ofen been observed, but appers to be caused by multiple interacting factors such as clay, silt, sand content and exchangeable cation balances (Roobroeck et al., 2021). Moreover, landscape positions often dictate crop fertilizer responses in highland farming systems (Amede et al., 2020). To address such complex interactions, it will likely be necessary to include additional geospatial information as well as rule-based algorithms in the existing SSNM solutions. A new 30-m resolution digital soil map is now free available for the entire content (Hengl et al., 2021), which should be explored for such purposes. These maps could provide soil information such as soil texture, acidity, Mn and Al toxicity, soil erosion, and soil crusts, and adjust soil fertility and nutrient management practices (e.g. liming for acidic soils, lower targeted yield for sandy soils) for integration with SSNM. Other examples exist on how simple, field-based observations on soil and landscape can be incorporated in making local fertilizer recommendations (White et al., 2000).

Additional challenges include limitations in: requisite capacity for use of available tools; resources for calibration, evaluation and validation exercises (see below); and knowledge on the usefulness of digital tools in developing SSNM among agricultural stakeholders, with the use of digital tools mostly limited to stakeholders in the research domain (MacCarthy et al., 2015).

Expanding the geographical reach of SSNM based digital tools requires resources for calibration, evaluation and validation of SSNM. As a first step for this expansion, there is need to estimate INS, which is required for calculating the amount of fertilizer nutrients to be added under the SSNM approach to enable effective utilization of existing nutrients. INS estimation has mostly been based on nutrient omission trials (Dobermann et al., 2003a), which requires at least two seasons to determine, presenting a bottleneck for scaling SSNM approach. This is because estimating INS based on soil properties using the first step of QUEFTS (Janssen et al., 1990) is also constrained by the limited soil testing capacity in smallholder farming systems in SSA and the poor predictive capability of commonly used soil tests (Schut and Giller, 2020). For example, attempts to estimate INS based on soil properties resulted in underestimation of cassava yields in Ghana and Togo (Ezui et al., 2017). However, the alternative approach used in NE to assess INS can potentially provide close estimates of INS (Pampolino et al., 2012). In this approach, INS is estimated based on site information like water availability (including the occurrence of flooding or drought), soil fertility proxies (soil texture, soil color, soil organic matter, P and K content (where available), historical use of organic resources, fertilizer inputs in previous cropping season, and farmers’ current yields. For nutrients such as N, it may also be possible to estimate the indigenous supply with the help of more mechanistic carbon and N mineralization models, as done in the Maize-N model (Setiyono et al., 2011). These INS estimation proxies need to be tested across regions and cropping systems before they can be adopted.

Weather forecast tools can also be used to improve fertilizer advisories. For example, an integrated decision support tool, WeRise (weather-rice-nutrient integrated decision support system) combines the use of local seasonal climate forecast models with ORYZA simulation model has been used to provide advise on optimum rice sowing dates for chosen varieties and nutrient management for rainfed lowland rice production systems in Southeast Asia (Hayashi et al., 2021). This integrated tool led to increased rice productivity and fertilizer recovery in rainfed rice cropping systems. Ensuring adherence to good agronomic practices is also important for warranting the performance of the recommendations and nutrient responses (Ravensbergen et al., 2021). For example, without adequate land development (e.g. bunding and levelling) in both rainfed lowland and irrigated rice systems and proper weed management, nutrient management practices such as SSNM would not work effectively (Becker and Johnson, 2001, Haefele et al., 2000, Ibrahim et al., 2021). Research is also needed to integrate and validate these different tools and approaches for more extensive evaluation in diverse and fully representative farming systems in SSA. Further, introduction of the science of decision support tools in agricultural training curricula at the tertiary level, policy support for the adoption of use of decision support tools in developing SSNM, and simplification of decision support tools to facilitate their use by non-scientific audiences, is required to enhance the application of SSNM (MacCarthy et al., 2015). However, it is important to co-develop any such decision support tools with continuous input by end users, from early prototype stages to rigorous evaluation in the field. The most difficult assumptions should be tested first.

This review focussed on three crops, rice, maize and cassava. As shown in Fig. 1, there was almost no linkage in research among those crops in SSA. So far, none of the SSNM decision support tools deal with more than one crop in SSA, yet the cropping systems in the region are diversified. Recently, the Excellence in Agronomy initiative (EiA; https://www.cgiar.org/initiative/11-excellence-in-agronomy-eia-solutions-for-agricultural-transformation/) was initially established among 10 CGIAR Research Centers and we expect that this establishment could help acceralating cross-learning among researchers working across different crops and cropping systems in developing new decision support tools, which can deal with multiple crops. Furthermore, the Excellence in Agronomy initiative strongly collaborates with public and/or private partners requesting specific agronomic innovations and having dissemination approaches. Demand-driven innovation in the Excellence in Agronomy initiative is meant to also improve the wider adoption of agronomic solutions for smallholder farmers.

## 5 Conclusions

This review shows that the SSNM (site-specific nutrient management) approach can provide robust nutrient management recommendations for rice, maize and cassava to smallholder farmers in SSA (sub-Saharan Africa) where soil fertility is highly variable. The SSNM approach offers a promising avenue for improving yield, profitability and N use efficiency for these crops compared to the farmer practice, and the local blanket fertilizer recommendations. Digital decision support tools such as RiceAdvice, Nutrient Expert and AKILIMO were developed and calibrated for SSA conditions and are freely available for millions of smallholders. However, SSNM research and dissemination of digital decision support tools in the region have been limited to few crops and geographies. As the SSNM approach and its challenges are quite similar among the three crops, cross-learning and joint efforts could enable to accelerate research and dissemination efforts. For wider adaptation of SSNM approaches in this continent, there is need to account for the effect of specific soil constraints on expected crop yield responses. In addition, the development of proxies to estimate INS, more resources for calibration, evaluation and validation, and functional and sustainable dissemination approaches through public and private sector channels can also enhance wide dissemination of SSNM. Integration of the SSNM digital decision support tools with remote sensing, weather forecast tools that estimate locally relevant conditions, and local prices can improve fertilizer recommendation and provide additional information on soil fertility, climate and economic uncertainty.

## Declaration of Competing Interest

The authors declare that they have no known competing financial interests or personal relationships that could have appeared to influence the work reported in this paper.
